# Nonarteritic anterior ischemic optic neuropathy and incidence of Parkinson’s disease based on a nationwide population based study

**DOI:** 10.1038/s41598-024-53196-9

**Published:** 2024-02-05

**Authors:** Jong Hyeon Ahn, Min Chae Kang, Jinyoung Youn, Kyung-Ah Park, Kyung-Do Han, Jin-hyung Jung

**Affiliations:** 1 Department of Neurology, Samsung Medical Center, Sungkyunkwan University School of Medicine, 81 Irwon-ro, Gangnam-gu, Seoul, 06351 Republic of Korea; 2 Neuroscience Center, Samsung Medical Center, Seoul, Republic of Korea; 3 Department of Ophthalmology, Samsung Medical Center, Sungkyunkwan University School of Medicine, 81 Irwon-ro, Gangnam-gu, Seoul, 06351 Republic of Korea; 4 Department of Statistics and Actuarial Science, Soongsil University, Seoul, Republic of Korea; 5 Samsung Biomedical Research Institute, Sungkyunkwan University School of Medicine, Suwon, Republic of Korea

**Keywords:** Epidemiology, Neurology, Optic nerve diseases

## Abstract

This study aimed to investigate the association between nonarteritic anterior ischemic optic neuropathy (NAION) and Parkinson's disease (PD) using a retrospective, nationwide, population-based cohort in South Korea. This study utilized data from the Korean National Health Insurance database, including 43,960 NAION patients and 219,800 age- and sex-matched controls. Cox proportional hazards regression models were used to assess the risk of developing PD in the NAION group compared to the control group after adjusting for various confounding factors. Subgroup analyses were conducted based on sex, age, and comorbidities. The incidence rate of PD was higher in the NAION group (1.326 per 1000 person-years) than in the control group (0.859 per 1000 person-years). After adjusting for confounding factors, the risk of developing PD was significantly higher in the NAION group (adjusted hazard ratio [aHR] 1.516, 95% confidence interval [CI] 1.300–1.769). Subgroup analyses did not reveal a significant difference in the risk of PD development based on sex, age, or comorbidities. This retrospective, nationwide, population-based cohort study revealed a significant association between NAION and an increased risk of developing PD in a South Korean population. The incidence rate of PD was observed to be higher in individuals diagnosed with NAION than in age- and sex-matched controls even after adjusting for potential confounding variables, with the risk being approximately 51.6% higher in the NAION group. Further research is necessary to elucidate the underlying pathophysiological mechanisms linking NAION to PD and to determine whether similar associations exist in other ethnic and geographical populations.

## Introduction

Nonarteritic anterior ischemic optic neuropathy (NAION) is the most common acute optic neuropathy in elderly patients^1^. NAION is a multifactorial disease that causes hypoperfusion in short posterior ciliary arteries or their tributaries supplying the optic nerve, resulting in optic nerve head ischemia^1–3^. The exact mechanisms underlying the development of optic disc ischemia in NAION are not fully understood yet. It remains unclear whether ischemia is caused by local arteriosclerosis with or without thrombosis, embolization from a distant source, systemic hypoperfusion, vasospasm, failure of autoregulation, or combinations of these factors^4,5^. It has been suggested that arterial hypotension might play a major role in the development of NAION and that cardiovascular risk factors such as diabetes mellitus (DM), hypertension (HTN), hyperlipidemia, anemia, obstructive sleep apnea, coagulopathies, and smoking are associated with an increased risk of NAION^5,6^.

Parkinson’s disease (PD) is a progressive neurodegenerative disease caused by accumulation of alpha-synuclein in the substantia nigra, although the exact mechanism remains unknown^7^. Individuals with PD not only show motor symptoms such as rigidity, bradykinesia, postural instability, and resting tremor, but also with a variety of non-motor symptoms including autonomic dysfunction^8^. Autonomic dysfunction associated with PD can lead to hypoperfusion and orthostatic hypotension (OH) which can cause episodic and recurrent systemic hypotension^9–11^. Those symptoms can emerge even in the early stage or prodromal stage of the disease^12–14^. These hypoperfusion and recurrent systemic hypotension potentially contribute to the development of NAION^6^. In addition, both NAION and PD share common vascular risk factors such as DM, HTN, and dyslipidemia^5,6,15–17^. Moreover, previous study has reported that peptic ulcer is found at higher rates in both NAION and PD patient groups than in age- and sex- matched controls^18^. Recently, there is growing evidence of a strong association between eye-related issues and PD. Not only have structural alterations in the retina and macula been observed in PD patients^19^, but also microvascular changes in the macula, particularly in those with OH, have been reported^20^. Given these shared pathomechanisms, it is crucial to further investigate the relationship between NAION and PD for a better comprehension of both conditions.

While numerous studies are ongoing concerning the correlation between neurodegenerative diseases (such as Alzheimer’s disease and PD) and ophthalmological neurodegenerative diseases (such as glaucoma and age-related macular disease)^21,22^, research specifically addressing a correlation between NAION and PD has not been reported yet. PD has been identified as a disease associated with vascular abnormalities and reduced perfusion^23^. Given that NAION is also a vascular disease, particularly characterized by occlusion of small vascular branches at the optic nerve head^3^, more so than other ophthalmic conditions, it is plausible to speculate a correlation between the two diseases. Thus, the aim of this study was to explore this relationship using an epidemiological approach. We focused on Korean adults and utilized the National Sample Cohort (NSC) database from the National Health Insurance Service (NHIS) of Korea to conduct this research.

## Methods

### Data source and study setting

The Korean National Health Insurance (NHI) is a health insurance system that provides coverage to approximately 97% of the Korean population, while the remaining 3% receive medical protection benefits. The NHIS maintains a database that contains information on medical facility usage, prescriptions, and diagnostic codes representing Korean Standard Classification of Diseases (KCD)-7 codes. These codes are based on the International Classification of Diseases (ICD)-10 codes with some modifications specific to Korean healthcare. Additionally, all beneficiaries aged 20 years or older have the opportunity to participate in the biennial health examination program, which includes a self-reported medical history, behavior, and laboratory tests^24^. This anonymized and de-identified database is considered representative of the Korean population. This study was approved by the Samsung Medical Center Institutional Review Board (IRB no. SMC 2020-09-050). The requirement for informed consent from individual patients was waived because data used were public and anonymized under confidentiality guidelines.

### Study population

**Figure 1:**
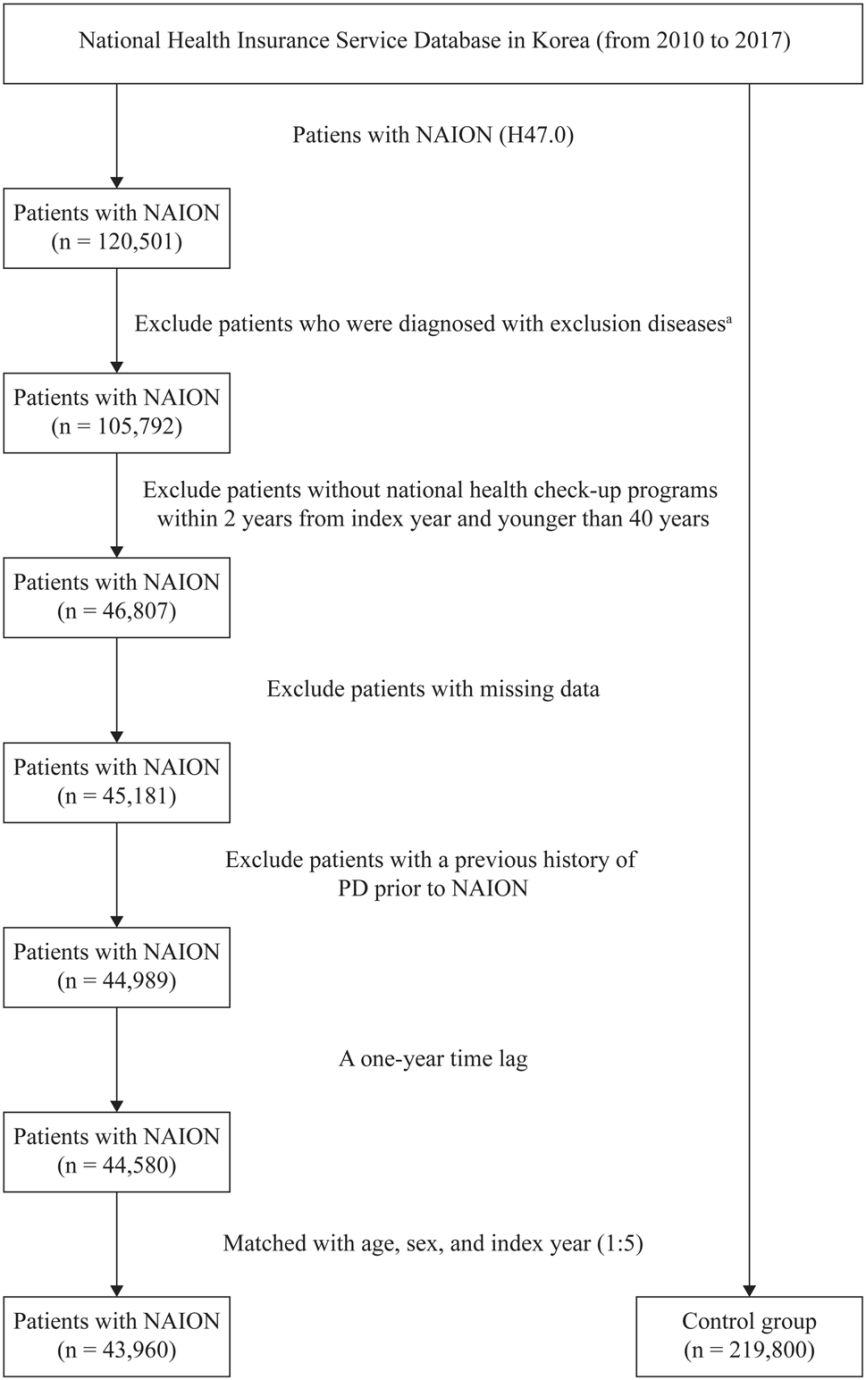
Flowchart showing the enrollment process of the current study cohort. NAION, nonarteritic anterior ischemic optic neuropathy; PD, Parkinson’s disease. ^a^Excluded diseases: trauma or brain conditions potentially leading to papilledema (S02.0-S09.9, G93.2, G93.5, G93.6, H47.1, I67.4), optic neuritis (H46.0); demyelinating diseases of the central nervous system including neuromyelitis optica (G36.0); multiple sclerosis (G35.0); other systemic connective tissue diseases (M35.3); giant cell arteritis (M31.5 and 31.6), primary malignant neoplasms of the eye and adnexa (C69, C72), meninges (C70), brain (C71), other parts of the central nervous system (C79); secondary malignant neoplasms of the brain and meninges (C79.3), other or unspecified parts of the nervous system (C79.4); benign neoplasms of the orbit (D31.6), meninges (D32), brain (D33), and other parts of the central nervous system (D35.2).

A study cohort consisting of individuals diagnosed with NAION (H47.0) per ICD-10 code from 2010 to 2017 was established. This cohort included individuals (n = 120,501) with a minimum of two outpatient visits or one hospitalization. Patients with a history of trauma or brain conditions potentially leading to papilledema (S02.0-S09.9, G93.2, G93.5, G93.6, H47.1, I67.4), those with optic neuropathies such as optic neuritis (H46.0) other than NAION, those with demyelinating diseases of the central nervous system including neuromyelitis optica (G36.0), those with multiple sclerosis (G35.0), those with other systemic connective tissue diseases (M35.3), and those with giant cell arteritis (M31.5 and 31.6) were excluded. Additionally, those with primary malignant neoplasms of the eye and adnexa (C69, C72), meninges (C70), brain (C71), and other parts of the central nervous system (C79), those with secondary malignant neoplasms of the brain and meninges (C79.3) or other or unspecified parts of the nervous system (C79.4), and those with benign neoplasms of the orbit (D31.6), meninges (D32), brain (D33), or other parts of the central nervous system (D35.2) were excluded (n = 105,792). Those who had undergone health checkups designed for individuals aged 40 years and older within two years prior to their initial NAION diagnosis were included (n = 46,807). Patients with missing data for covariables were excluded (n = 45,181). A one-year time lag was implemented to prevent causal reversal scenarios. As a result, 44,580 patients were deemed eligible from the initial population. Following a sex-age matching process at a 5:1 ratio, the study population included 43,960 NAION patients and 219,800 matched controls (Fig. 1).

### Covariates

Baseline comorbidities were evaluated using patients' medical histories, clinical records, and pharmacy codes according to the ICD-10-CM. HTN was defined as a blood pressure of 140/90 mmHg or higher, or at least one prescription for anti-hypertensive medication within a year, with ICD-10-CM codes ranging from I10–I13 and I15. DM was determined by a fasting glucose level of 126 mg/dL or higher or a minimum of one prescription claim within a year. Dyslipidemia was diagnosed with a total cholesterol level of 240 mg/dL or higher or at least one antihyperlipidemic medication prescription within a year under ICD-10 code E78. Chronic kidney disease (CKD) was identified by an estimated glomerular filtration rate (eGFR) of less than 60 ml/min/1.73 m^2^. Standardized self-reported questionnaires were employed to gather general health behavior and lifestyle data during National health examination. Information on smoking status, alcohol consumption, and regular exercise was collected through those questionnaires. Obesity was defined as a body mass index of 25 kg/m^2^ or higher. Low-income level was designated as the lowest one-fifth of the entire population. Peptic ulcer was diagnosed under ICD-10 code K27 within a year.

### Study outcome and follow-up

The primary outcome was the incidence of newly diagnosed PD cases during the follow-up period. In South Korea, all patients diagnosed with PD are registered in the Rare Intractable Disease (RID) program. This program was implemented by the Korean government in 2006 to address rare intractable diseases, including PD. One of the objectives of this program is to reduce co-payment burden for registered PD patients by up to 10% from the usual 30%. Consequently, incident PD can be identified by both the ICD-10 code for PD (G20) and the registration code (V124) of the RID program. The V124 code is specifically assigned to patients diagnosed with PD by a neurologist or neurosurgeon. It is not used for secondary parkinsonism conditions such as vascular parkinsonism, drug-induced parkinsonism, traumatic brain injury, post-infectious parkinsonism, or hypoxic brain damage. The study cohort was followed up after a 1-year lag period from the date of cohort entry (first diagnosis date for NAION patients or matched index date for control subjects) to the earliest date of PD diagnosis or the end of follow-up (December 31, 2019), whichever occurred first.

### Statistical analysis

Baseline characteristics were compared between groups using Student's t-test or analysis of variance for continuous variables and chi-square test for categorical variables. Data are presented as numbers and percentages for categorical variables or mean ± standard deviation for continuous variables. Cases with NAION were matched to controls based on the year of NAION diagnosis. Five matched controls were chosen for each NAION case. The index date was matched between NAION cases and controls. The incidence of PD associated with prevalent NAION was assessed by Cox proportional hazards regression using crude and multivariable-adjusted models. Five models were constructed to account for various confounding factors. Model 1 was unadjusted. Model 2 was adjusted for age and sex. Model 3 was additionally adjusted for smoking status, alcohol consumption, regular exercise, and income level. Model 4 additionally considered medical conditions such as obesity, DM, HTN, dyslipidemia, and CKD. Model 5 was further adjusted to include peptic ulcer, which had previously been identified as a commonality between NAION and PD. Hazard ratios (HRs) with 95% confidence intervals (CIs) were calculated. Kaplan–Meier curve was employed to calculate cumulative incidence of PD in each group. Stratified analyses were conducted by sex and age (40–65 years and ≥ 65 years, considering the mean age of onset for PD), demographics, and presence of each comorbidity. Propensity score matching (PSM) was used to control confounding factors between NAION and control groups. The propensity of being in the NAION group was estimated with a logistic regression model, including all variables from our research database. These variables included age, sex, income, body mass index, smoking, drinking, regular exercise, diabetes, hypertension, dyslipidemia, chronic kidney disease, and peptic ulcer. Each patient in the control group was matched to one patient in NAION group (1:1 matching). Comparison of baseline characteristics in the NAION group and control group was performed based on absolute standardized difference (ASD). An ASD value of less than or equal to 0.1 (10%) signified a minimal disparity between the two groups for each covariate^25^. All statistical analyses were performed using the SAS statistical package version 9.4 (SAS Institute, Cary, North Carolina, USA) and a *p* value < 0.05 was deemed statistically significant.

### Ethical approval

All methods and experiments were designed and performed in accordance with the Declaration of Helsinki and relevant guidelines and regulations provided by the policies of Nature Portfolio journals. This research was reviewed and approved by the Institutional Review Board (IRB) of Samsung Medical Center (IRB no. SMC 2020-09-050).

## Results

### Baseline characteristics and the study population

Clinical characteristics and demographics of patients are summarized in Table 1. The mean age of NAION patients was 61.7 ± 10.0 years. There were 44.3% males. The mean follow-up duration was 3.7 ± 2.0 years. The NAION group had a higher prevalence of those with a lower income, a lower prevalence of obesity, and lower rates of current smoking and alcohol consumption. The proportion of people who engaged in regular exercise was higher in the NAION group. In addition, the NAION group had higher prevalence of DM, HTN, dyslipidemia, CKD, and peptic ulcer than the control group (Table 1). Baseline demographics of subjects after PSM are shown in Supplementary Table S1.

**Table 1:** Baseline characteristics of subjects.

	Total (N = 263,760)	NAION (N = 43,960)	Controls (N = 219,800)	*p* value
Age, years	61.7 ± 10.9	61.7 ± 10.9	61.7 ± 10.9	> 0.999
≥ 65 years	110,838 (42.0)	18,473 (42.0)	92,365 (42.0)	> 0.999
Sex, male, n (%)	116,718 (44.3)	19,453 (44.3)	97,265 (44.3)	> 0.999
Follow-up duration, years	3.7 ± 2.0	3.7 ± 2.0	3.7 ± 2.0	0.551
Income, lower 20%	55,986 (21.2)	8,507 (19.4)	47,479 (21.6)	< 0.001
Obesity, n (%)	95,925 (36.4)	15,183 (34.5)	80,742 (36.7)	< 0.001
Current Smoker, n (%)	38,924 (14.8)	5,331 (12.1)	33,593 (15.3)	< 0.001
Drinking, n (%)	89,601 (34.0)	14,280 (32.5)	75,321 (34.3)	< 0.001
Regular exercise, n (%)	57,005 (21.6)	9,964 (22.7)	47,041 (21.4)	< 0.001
DM, n (%)	45,735 (17.3)	9,199 (20.9)	36,536 (16.6)	< 0.001
HTN, n (%)	120,374 (45.6)	20,872 (47.5)	99,502 (45.3)	< 0.001
Dyslipidemia, n (%)	99,374 (37.7)	18,337 (41.7)	81,037 (36.9)	< 0.001
CKD, n (%)	20,251 (7.7)	3,587 (8.2)	16,664 (7.6)	< 0.001
Peptic ulcer, n (%)	24,017 (9.1)	4,565 (10.4)	19,452 (8.9)	< 0.001

Data are presented as mean ± standard deviation.

*NAION* nonarteritic anterior ischemic optic neuropathy, *DM* diabetes mellitus, *HTN* hypertension, *CKD* chronic kidney disease.

### Risk of PD in NAION patients vs. matched controls

**Figure 2:**
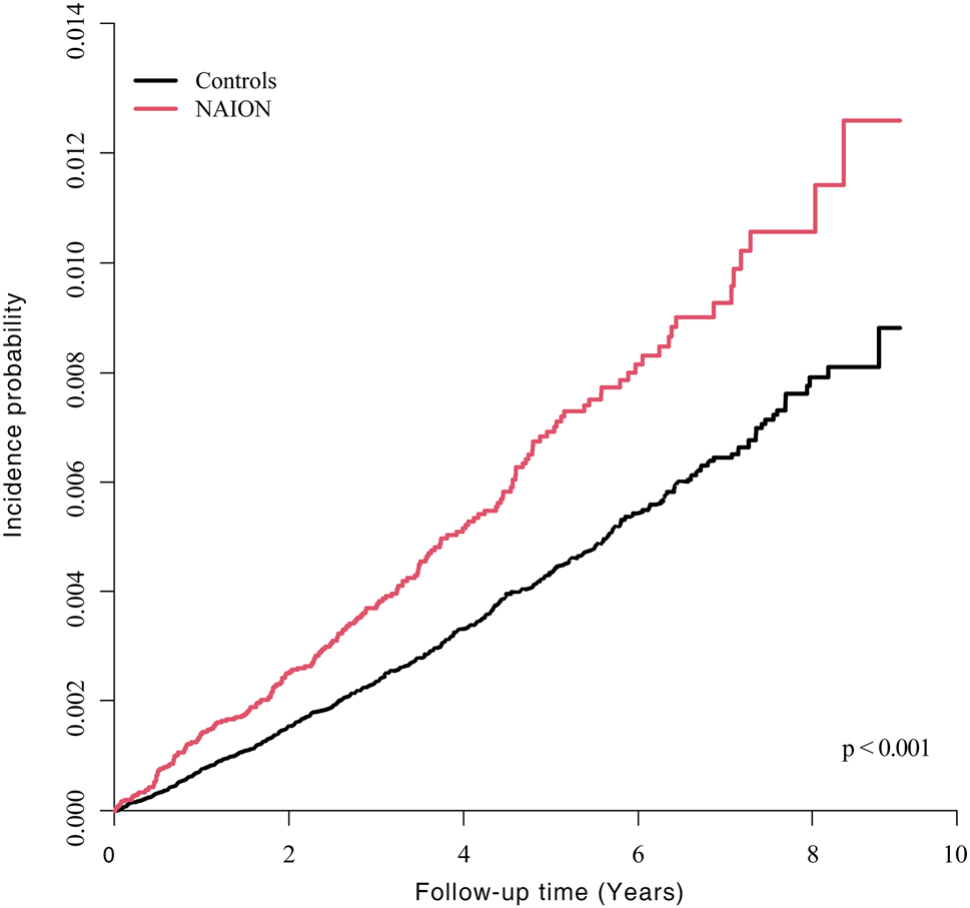
Kaplan–Meier curves of Parkinson’s disease by NAION. NAION, nonarteritic anterior ischemic optic neuropathy; Follow-up time (years), the time since the index date in the Kaplan–Meier survival analysis. The NAION group had a lower PD disease-free survival rate than the control group.

During the follow-up period of 3.7 ± 2.0 years, PD was diagnosed in 214 of 43,960 NAION patients. The incidence rate of PD was 1.326 per 1000 person-years in the NAION group, exceeding the rate of 0.859 per person-years in the control group. In model 5, NAION patients exhibited a significantly higher risk of developing PD than the control group (aHR 1.514, 95% CI 1.298–1.765) (Table 2 and Fig. 2). In PSM models, even after matching ratios of all covariates, the risk of developing PD in NAION patients remained significantly higher compared to controls (Table 3).

**Table 2:** Incidence rates of Parkinson’s disease in nonarteritic anterior ischemic optic neuropathy patients.

	N	Events	Person-years^a^	Incidence Rate (per 1000 PY)^b^	Adjusted hazard ratio (95% CI)				
					Model 1	Model 2	Model 3	Model 4	Model 5
Comparison between NAION and control group									
Control	219,800	694	808,202.3	0.859	1.00 (ref.)	1.00 (ref.)	1.00 (ref.)	1.00 (ref.)	1.00 (ref.)
NAION	43,960	214	161,368.9	1.326	**1.545 (1.325, 1.801)**	**1.542 (1.323, 1.798)**	**1.525 (1.308, 1.778)**	**1.516 (1.3, 1.769)**	**1.514 (1.298, 1.765)**

Model 1 was unadjusted.

Model 2 was adjusted for age and sex.

Model 3 was adjusted for age, sex, smoking, alcohol drinking, regular exercise, and income.

Model 4 was adjusted for age, sex, smoking, alcohol drinking, regular exercise, income, diabetes, hypertension, dyslipidemia, body mass index, and chronic kidney disease.

Model 5 was adjusted for age, sex, smoking, alcohol drinking, regular exercise, income, diabetes, hypertension, dyslipidemia, body mass index, chronic kidney disease, and peptic ulcer.

Significant values are in [bold].

*NAION* nonarteritic anterior ischemic optic neuropathy, *PY* person-year, *CI* confidence interval.

^a^Person-years was calculated as observed time interval*number of observed subjects.

^b^Incidence rates in 1000 person-years.

**Table 3:** Incidence rates of Parkinson’s disease in nonarteritic anterior ischemic optic neuropathy patient after propensity score matching.

	N	Events	Person-years^a^	Incidence rate (per 1000 PY)^b^	Adjusted hazard ratio (95% CI)	
					Model 1	Model 2
Comparison between NAION and control group						
Control	43,960	126	161,604.8	0.780	1.00 (ref.)	1.00 (ref.)
NAION	43,960	214	161,368.9	1.326	**1.701 (1.365, 2.119)**	**1.686 (1.353, 2.101)**

Model 1 was propensity score matched-model, matched for age, sex, smoking, alcohol drinking, regular exercise, income, diabetes, hypertension, dyslipidaemia, body mass index, and chronic kidney disease.

Model 2 was propensity score matched-model, matched for age, sex, smoking, alcohol drinking, regular exercise, income, diabetes, hypertension, dyslipidaemia, body mass index, chronic kidney disease, and peptic ulcer.

Significant values are in [bold].

*NAION* nonarteritic anterior ischemic optic neuropathy, *PY* person-year, *CI* confidence interval.

^a^Person-years was calculated as observed time interval*number of observed subjects.

^b^Incidence rates in 1000 person-years.

### Risk of PD by age, sex, and comorbidities

Subgroup analysis showed no marked differences in the risk of developing PD based on sex, age group, demographic factors, or comorbidities between the two groups. Results revealed that the risk of PD development in patients NAION did not show a significant association with the presence of DM, HTN, dyslipidemia, or CKD when compared with the control group.

## Discussion

In this retrospective, nationwide, population-based cohort study, the development of PD in the Korean population was found to be higher in the NAION group than in the non-NAION control group even after adjusting for age, sex, smoking status, drinking amount, regular exercise, obesity, the status of income, and comorbidities such as DM, HTN, dyslipidemia, CKD, and peptic ulcer. There was no significant difference in PD risk based on sex, age, demographics, or comorbidities among NAION patients.

Our results revealed a 1.5-fold higher risk of developing PD in patients with NAION compared to the non-NAION control group, thereby supporting a potential relationship between these two conditions. Although prior research studies have shown associations between PD and ophthalmological impairments such as thinning of the inner retina, progressive parafoveal thinning, fovea avascular zone remodeling, and retinal microvascular damage^19,20,26^, the distinct association between NAION—the most common ischemic optic neuropathy in the elderly—and the risk of developing PD has not been intensively explored. Our study addressed this knowledge gap. It sheds light on the potential link between NAION and PD.

One notable finding in our study was the consistent association between NAION and PD, which remained significant even after adjusting for multiple confounding factors. Our analysis meticulously considered diverse demographics and clinical factors, including age, sex, income status, obesity, smoking status, alcohol consumption, regular exercise, and comorbidities such as DM, HTN, dyslipidemia, CKD, and peptic ulcer. This trend persisted in PSM models even after matching the distribution of other factors. In addition, our subgroup analyses according to demographic factors and comorbidities did not yield significant differences. This suggests that the observed association between NAION and PD is unlikely to be influenced by these underlying health conditions. These findings, in conjunction with the adjusted model and subgroup analyses, support the premise of a direct association between NAION and PD, independent of confounding factors or other comorbidities.

The mechanism by which the risk of PD increases in patients with NAION is not yet clearly established. However, various potential mechanisms can be considered.

Previous studies have posited the pathogenic mechanism of NAION as transient profound hypoperfusion or nonperfusion within the optic nerve, leading to ischemic damage^5,6,27–29^. According to many studies in PD patients, there are changes in white matter related to the progression of the disease, which are known to involve venous structural and flow abnormalities^30^. Further, it has been reported that compared to healthy controls, PD patients show vascular abnormalities and reduced perfusion^23^. Additionally, chronic cerebral hypoperfusion has been identified as a risk factor for the exacerbation of symptoms such as memory decline and motor dysfunction in PD patients^31,32^. In other words, hypoperfusion, which is implicated in the onset of NAION, can act as a major risk factor in the onset and progression of PD.

Additionally, OH, a common non-motor symptom in PD with a prevalence of 30%, is present in 2.3% of prodromal PD patients^11,13^. Our prior research has demonstrated an association between PD patients with OH and microvascular damage in central retinal microvasculature^20^. However, research on the association between OH and NAION is still insufficient, indicating the need for further studies. There has been only one case report of bilateral blindness due to ischemic optic neuropathy associated with OH^33^. Additional research is necessary. The current study did not explicitly evaluate the presence of OH or perfusion state. The mutual association with systemic hypotension and hypoperfusion in both NAION and PD underscores the necessity for further investigation into the role of this common risk factor.

Previous studies have reported that the risk of developing PD is significantly increased in patients with glaucoma, one of the progressive optic neuropathies^34^. While the pathogenesis is not yet clearly established, there is a hypothesis that mitochondrial dysfunction leading to oxidative stress caused by reactive oxygen species may damage retinal ganglion cells and dopaminergic neurons, a common mechanism through which both glaucoma and PD may be induced^35–37^. Similarly, oxidative stress has been reported to be associated with various optic neuropathies besides glaucoma^38^. There are reports indicating that such oxidative stress can induce vasoconstriction, affecting microcirculation^39^. In NAION, oxidative stress due to mitochondrial alteration has also been reported as a potential risk factor^40,41^. This suggests a possible mechanism for the development of PD in NAION, although further research is needed in this area.

NAION and PD share several common vascular risk factors. The optic nerve is a part of the white-matter tract of the central nervous system. Thus, NAION is considered a type of white matter stroke^42^. Consequently, well-established risk factors for NAION include traditional cardiovascular risk factors^43–45^ such as DM^44^, HTN^44,46^, dyslipidemia^44^, obstructive sleep apnea^47,48^, and coronary artery disease^44,49^ in addition to typical optic disc morphology^49^. These cardiovascular risk factors are also associated with an increased risk of PD. Emerging PD research suggests that patients with these cardiovascular risk factors including DM^50,51^, HTN^52^, obstructive sleep apnea^53^, and coronary artery disease^54^ are at a higher risk of developing PD compared to those without these diseases. These shared risk factors might contribute to the observed elevation of PD risk in NAION patients. Considering these overlapping vascular risk factors, we can gain a better understanding of the potential relationship between NAION and PD. This highlights the importance of investigating these common risk factors and their underlying mechanisms to elucidate the link between the two diseases. However, in this study, the primary focus was not on the analysis of risk factors, but on the association between the two diseases. As such, a multivariable analysis of risk factors was not conducted. Further analysis is needed to explain the causal relationship between the two diseases in terms of common risk factors.

In the NAION patient group included in this study, modifiable lifestyle-related risk factors, such as obesity, smoking, and drinking, were found to be lower than in the control group, while the rate of regular exercise was higher. This is different from results of many previous studies showing that smoking, a well-known vasculopathic risk factor, plays a significant role in NAION^55–59^, even though the finding of smoking as a risk factor for NAION remains controversial^60^. This may suggest that observed lower levels of metabolic risk factors could be due to efforts of individuals to reduce these factors. Additionally, in studies like this, where a cross-sectional comparison is made with controls, accurately comparing actual proportions can be challenging. Furthermore, smoking has been known as a protective factor against the development of PD in previous studies^61–63^. Therefore, the lower smoking rate in NAION patients included in this study compared to controls, might have contributed to an increased risk of developing PD.

This study has several limitations. Firstly, its retrospective design hindered the establishment of a causal relationship between NAION and PD. Prospective studies are needed to address this. Secondly, the reliance on administrative healthcare data could introduce potential biases, including misclassification or underreporting of NAION and PD cases, which could impact the accuracy of our results. Also, in the NHIS data disease classification system, specific subcategories under H47.0 are not counted separately, which can be considered a limitation of this study. However, among subcategories of H47.0, ischemic optic neuropathy corresponding to the code H47.01, accounts for the majority. The prevalence of other subcategory diseases is not high. Therefore, the margin of error is expected to be minimal. Additionally, the lack of detailed clinical information such as disease severity and specific treatments limited our ability to explore confounding factors and underlying mechanisms. Moreover, the study's focus on the Korean population might limit the generalizability of our findings to other ethnicities and populations. Replication in diverse populations is necessary for validation. Lastly, although we adjusted for several confounding factors, residual confounding and unmeasured variables might still influence study results. Further research with prospective design and comprehensive data collection is needed to overcome these limitations and gain a deeper understanding of the association between NAION and PD.

In conclusion, our study furnishes evidence for an increased risk of developing PD in patients suffering from NAION. To the best of our knowledge, there has been no study elucidating a direct association between NAION and PD. Additionally, the significance of this study lies in examining the association between the two diseases themselves, controlling for various covariates. Even after adjusting for different covariates, the risk of developing PD in NAION patients was 1.5 times higher compared to controls. Gaining insight into the association between NAION and PD bears significance for both ophthalmological and neurological healthcare, potentially leading to improved management strategies and promoting early detection of PD in NAION patients. Further research studies, including prospective studies and experimental investigations, are warranted to deepen our understanding of the pathophysiological mechanisms underlying their association and explore potential therapeutic interventions targeting common pathways.

### Supplementary Information

Supplementary Table S1.

## Data Availability

The data supporting the findings of this study are available from the corresponding author upon request. The data are not publicly available due to privacy or ethical restrictions.
