# Supplementary material for: Nonarteritic anterior ischemic optic neuropathy and incidence of Parkinson’s disease based on a nationwide population based study
Source: Sci Rep. 2024 Feb 5;14:2930. doi: 10.1038/s41598-024-53196-9 (PMC10844599; doi:10.1038/s41598-024-53196-9)
Supplement: Supplementary file 1 — Supplementary Table S1. [file 41598_2024_53196_MOESM1_ESM.docx]

**Supplementary Table S1. Baseline characteristics of subjects after propensity score matching**

|  | **NAION**  **(N = 43,960)** | **Controls**  **(N = 43,960)** | ASD |
| --- | --- | --- | --- |
| Age, years | 61.7 ± 10.9 | 61.7 ± 10.9 | 0.004 |
| ≥ 65 years | 18,473 (42.0) | 18,561 (42.2) | 0.004 |
| Sex, male, n (%) | 19,453 (44.3) | 19,469 (44.3) | 0.001 |
| Income, lower 20% | 8,507 (19.4) | 8,284 (18.8) | 0.013 |
| Obesity, n (%) | 15,183 (34.5) | 15,163 (34.5) | 0.001 |
| Current Smoker, n (%) | 5,331 (12.1) | 5,171 (11.8) | 0.011 |
| Drinking, n (%) | 14,280 (32.5) | 14,339 (32.6) | 0.003 |
| Regular exercise, n (%) | 9,964 (22.7) | 9,916 (22.6) | 0.003 |
| DM, n (%) | 9,199 (20.9) | 9,015 (20.5) | 0.010 |
| HTN, n (%) | 20,872 (47.5) | 20,867 (47.5) | < 0.001 |
| Dyslipidemia, n (%) | 18,337 (41.7) | 18,289 (41.6) | 0.002 |
| CKD, n (%) | 3,587 (8.2) | 3,280 (7.5) | 0.026 |
| Peptic ulcer, n (%) | 4,565 (10.4) | 3,971 (9.0) | 0.046 |

NAION, Nonarteritic anterior ischemic optic neuropathy; ASD, absolute standardized mean difference; DM, Diabetes mellitus; HTN, hypertension CKD, Chronic kidney disease.

Data were presented with mean ± standard deviation.
